# Complement-Mediated Thrombotic Microangiopathies With Predominant Renal Involvement in a Patient With Lupus Nephritis

**DOI:** 10.7759/cureus.93685

**Published:** 2025-10-02

**Authors:** Aikya Manchikalapati, Paola Zinser Peniche, Nicoletta C Machin

**Affiliations:** 1 Internal Medicine, Gandhi Medical College and Hospital, Hyderabad, IND; 2 Division of General Internal Medicine, University of Pittsburgh Medical Center, Pittsburgh, USA; 3 Division of Hematology and Oncology, University of Pittsburgh Medical Center, Pittsburgh, USA

**Keywords:** complement-mediated thrombotic microangiopathy, lupus nephritis, systemic lupus erythematosus, thrombocytopenia, thrombotic microangiopathies

## Abstract

Complement-mediated thrombotic microangiopathies (cmTMAs) typically present with thrombocytopenia, microangiopathic hemolytic anemia, and microvascular end-organ damage. Due to an overlapping immune-mediated picture, cmTMAs are often observed in patients with systemic lupus erythematosus (SLE) and lupus nephritis (LN). We present a unique case of LN with cmTMAs predominantly involving the kidneys in the absence of persistent thrombocytopenia. A 20-year-old male with SLE experienced an unwitnessed syncopal event at his home, prompting hospitalization. Initial work-up was consistent with an active lupus flare with multisystem involvement. Despite initial treatment with pulse-dose steroids, he deteriorated with worsening renal function and transfusion-dependent anemia. Blood parameters showed evidence of hemolysis, although platelet counts remained stable and within normal limits throughout the admission. Concern for renal involvement and an atypical clinical presentation prompted a renal biopsy, which revealed immune complexes with evidence of thrombotic microangiopathy. Eculizumab and later ravulizumab were employed as part of his ongoing treatment plan, which resulted in rapid resolution of hemolysis followed by gradual renal recovery over eight weeks. Scientific literature supports the use of terminal complement inhibitors in the treatment of cmTMAs. This report adds to the emerging literature on cmTMAs limited to the kidneys and explores the efficacy of C5 complement inhibition in such patients.

## Introduction

Lupus nephritis (LN) is the renal manifestation of systemic lupus erythematosus (SLE) and presents with a wide range of microvascular renal lesions. This is attributed to the activation and dysfunction of endothelial cells and immune system dysfunction ​[1]. Thrombotic microangiopathies (TMA) are defined as a triad of thrombocytopenia, microangiopathic hemolytic anemia, and acute kidney injury​ [2]. Complement-mediated TMA (cmTMA) can occur in the context of genetic or acquired defects in complement proteins (primary cmTMA) or in association with other disease processes like autoimmunity, medications, malignancy, and infection (secondary cmTMA). Dysregulation of the classical and alternative complement pathways leads to uncontrolled activation of the membrane attack complex, causing endothelial injury, formation of microthrombi, and tissue ischemia​ [3].  

While systemic thrombotic microangiopathies are well-established in existing literature, predominant renal lesions are less frequently reported. The reliance on histopathological diagnosis, infrequent clinical presentation, and symptom overlap likely contribute to the infrequent reporting and identification of renal TMA in LN. Pathological study is not a part of routine evaluation in patients with LN; however, the emergence of new literature may prompt a shift in the diagnostic paradigm.  

First-line treatment for LN is with corticosteroids (pulse dose and maintenance dose), hydroxychloroquine, and immune-modulatory therapy (mycophenolate mofetil, cyclophosphamide, azathioprine, methotrexate, and more). Plasma exchange and intravenous immunoglobulin (IVIG) use is generally reserved for refractory cases. In patients with concomitant cm-TMA, clinical evidence reveals that standard therapies are largely inefficacious, and the use of terminal complement inhibitors may demonstrate more favourable outcomes with a significant improvement in renal function and hematological parameters ​[4].   

## Case presentation

**Figure 1:**
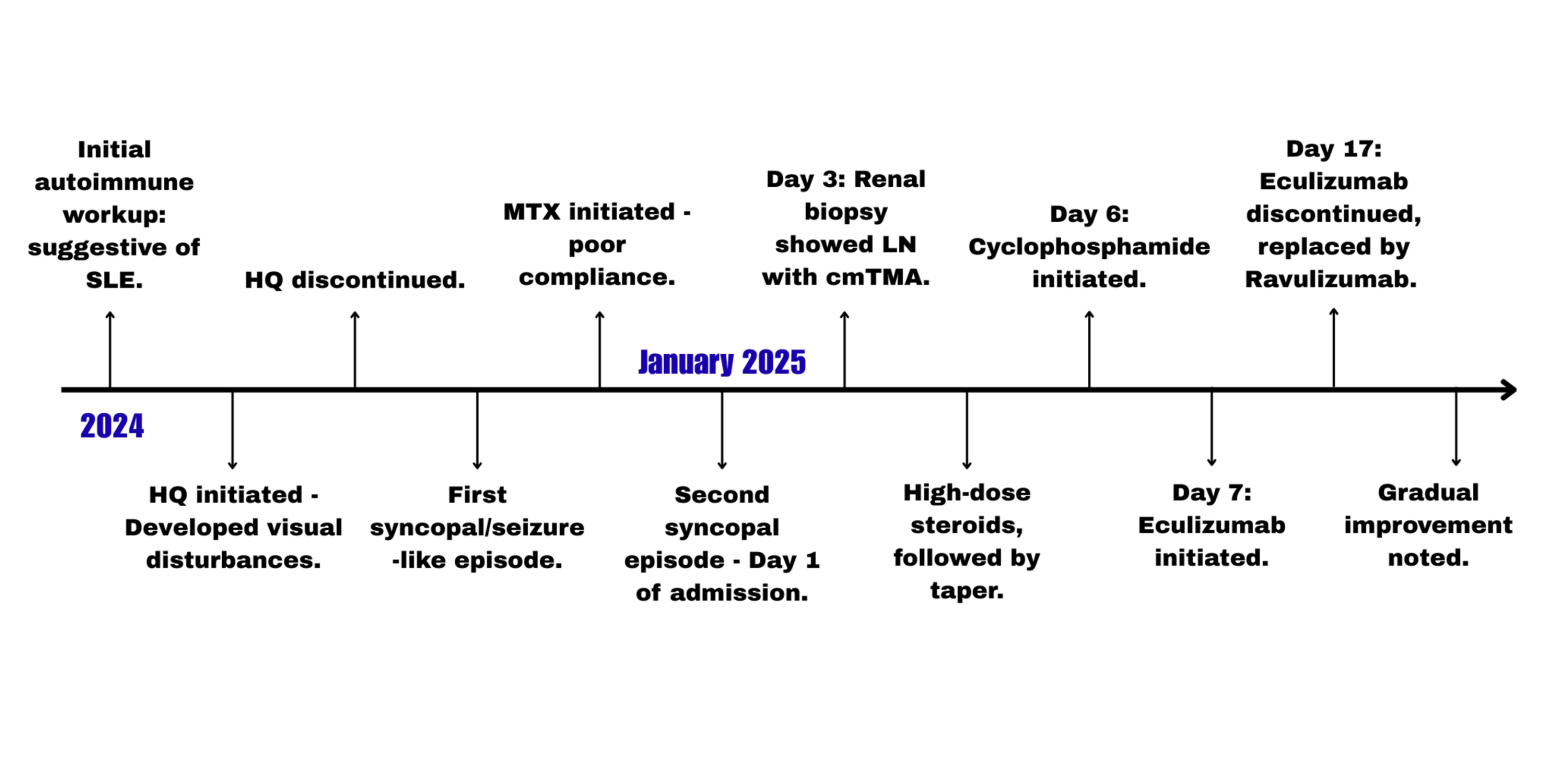
Early disease course and important events. HQ: hydroxychloroquine, MTX: methotrexate, SLE: systemic lupus erythematosus, LN: lupus nephritis, cmTMA: complement-mediated thrombotic microangiopathy

We describe a 20-year-old man with SLE who was hospitalized in January 2025 following an unwitnessed syncopal episode. His autoimmune workup had been initiated in early 2024 owing to symptoms including alopecia, cutaneous lesions, and inflammatory arthritis (refer to Figure 1 for early disease course). Laboratory evaluation at that time revealed a positive antinuclear antibody (ANA), elevated anti-double-stranded DNA (dsDNA), and anti-Sjögren's syndrome type A (SSA) antibodies, supporting a diagnosis of SLE. He was started on hydroxychloroquine, which was discontinued shortly thereafter when he developed visual disturbances. Within days of stopping the medication, he experienced his first syncopal/seizure-like episode. Methotrexate was subsequently added to address worsening arthritis, though he remained poorly compliant. He remained seizure- and syncope-free until the January 2025 event, which prompted his current hospitalization. Details of his hospital course are outlined below. 

**Figure 2:**
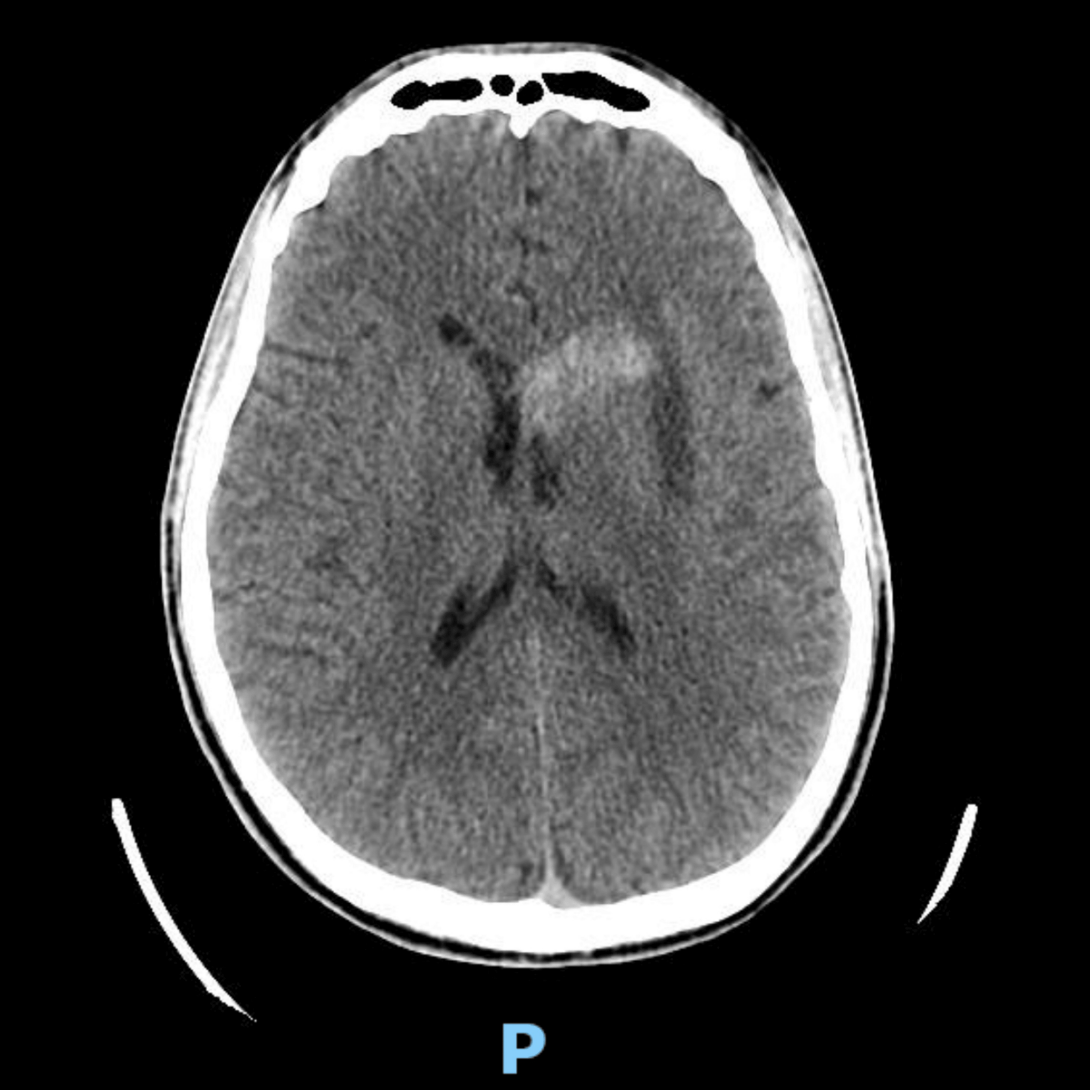
Head CT Non-contrast axial CT of the head showing a hyperdense hemorrhagic transformation of an acute infarct in the left basal ganglia with extension into the lateral ventricle, consistent with decompressive intraventricular hemorrhage.

On the day of the episode, the patient reported feeling lightheaded while in the bathroom and subsequently collapsed to the floor. He denied losing consciousness or sustaining any head trauma. He was evaluated at a local emergency department, where he endorsed worsening headache. His systolic blood pressure was elevated, reaching the 170s mmHg, though he remained afebrile and otherwise hemodynamically stable. Non-contrast head CT demonstrated a stable acute infarct with hemorrhagic transformation involving the basal ganglia and ventricles (Figure 2). Initial labs were notable for a hemoglobin of 5 g/dL and a platelet count of 86,000/μL. He received two units of packed red blood cells and platelets, with appropriate post-transfusion response - hemoglobin improved to 8.7 g/dL and platelets to 176,000/μL. Once stabilized, he was transferred to the UPMC Presbyterian Emergency Department. Admission labs are detailed in Table 1. 

**Table 1:** Laboratory investigations on admission. WBC: white blood cells, Hg: hemoglobin, HCT: hematocrit, MCV: mean corpuscular volume, PLT: platelet count, LDH: lactate dehydrogenase, PT: prothrombin time, INR: international normalised ratio, aPTT: activated partial thromboplastin time, Na: serum sodium, Cl: serum chloride, BUN: blood urea nitrogen, Cr: serum creatinine, Fe: serum iron, TIBC: total iron binding capacity, Fe saturation: percentage iron saturation.

	Patient Values	Reference Range (Units)
WBC	10.4	4.5 - 11.0 x 10³/µL
Hg	8.7	13.5 - 17.5 g/dL
HCT	25.4	41% - 53%
MCV	84.3	80 - 100
PLT	176	150 - 450 x 10³/µL
Reticulocytes	2.5	0.4% - 2.4%
LDH	543	140 - 280 U/L
Haptoglobin	<30	30 - 200 mg/dL
PT	13.8	10 - 13 seconds
INR	1.1	0.9 - 1.1
aPTT	24.7	25 - 35 seconds
Fibrinogen	413	200 - 400 mg/dL
Na	139	135 - 145 mmol/L
K	5.3	3.5 - 5.0 mmol/L
Cl	113	98 - 106 mmol/L
BUN	98	7 - 20 mg/dL
Cr	3.9	0.6 - 1.2 mg/dL
Calcium (Ca)	7.1	8.5 - 10.5 mg/dL
Magnesium (Mg)	2.4	1.7 - 2.2 mg/dL
Phosphorus	8.8	2.5 - 4.5 mg/dL
Fe	103	45 - 182 µg/dL
TIBC	202	250 - 420 µg/dL
Fe saturation	51	25-50%
Ferritin	935	10 - 282 ng/mL
Total bilirubin	0.5	0.3-1.5 mg/dL

**Figure 3:**
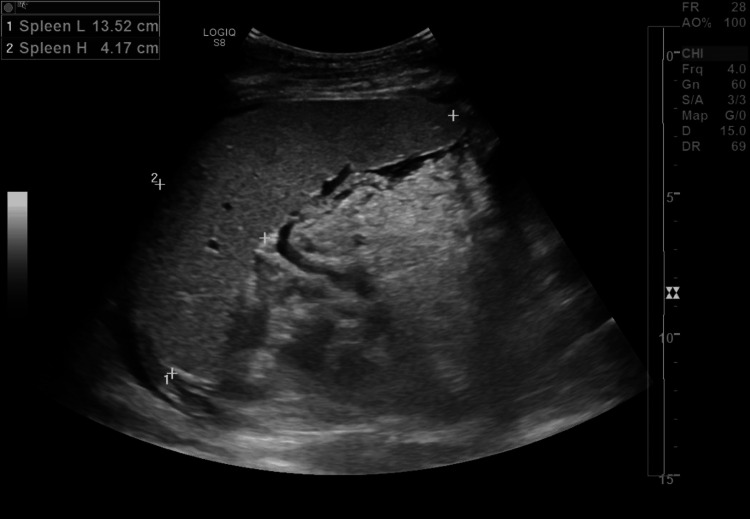
Abdominal ultrasound Spleen at the upper limit of normal size

The patient was admitted to the intensive care unit, and Hematology was consulted for evaluation of anemia and thrombocytopenia. Peripheral blood smear revealed more than eight schistocytes per high-power field, along with mature lymphocytes and no evidence of promyelocytes. Hemolytic workup showed haptoglobin <30 mg/dL, lactate dehydrogenase (LDH) of 543 U/L, and a reticulocyte count of 2.5%, raising concern for microangiopathic hemolytic anemia (MAHA). The presence of schistocytes, low haptoglobin, and elevated LDH supported this suspicion. However, an ADAMTS13 activity level of 48 IU/dL made thrombotic thrombocytopenic purpura (TTP) unlikely. Testing for antiphospholipid syndrome (APS), including IgG and IgM anticardiolipin and anti-beta 2 glycoprotein I antibodies, was negative, and there was no clinical or radiographic evidence of arterial or venous thrombosis. Abdominal ultrasound demonstrated borderline splenomegaly (Figure 3). Disseminated intravascular coagulation (DIC) was considered but ruled out based on normal coagulation studies and stable fibrinogen levels. To assess for an immune-mediated hemolytic process, a direct antiglobulin test was performed, and complement levels were checked. Additional rheumatologic investigations are summarized in Table 2. 

**Table 2:** Additional rheumatological Investigations dsDNA: double-stranded DNA, ANA: antinuclear antibodies, SS-A: Sjogren’s syndrome related antigen A, SS-B: Sjogren’s syndrome related antigen B, SM: smooth muscle, RNP: U1 ribonucleoprotein, GBM: glomerular basement membrane antibodies

	Patient values		Reference Range	Comment
	Day 2	Day 15		
Anti dsDNA	148	37	<10IU/ml	Positive
ANA Titre	1:320	1:1280	<1:40: Neg 1:40-1:80: Low >1:80: Elevated	Positive
Anti SS-A	3.9		<1.9 AI	Positive
Anti SS-B	0.2		<1.9 AI	Negative
Anti SM Antibodies	>8.0		<1.9 AI	Positive
Anti SM/RNP antibodies	5.0		<1.9 AI	Positive
RNP antibodies	1.3		<1.9 AI	Negative
Complement C3 levels	41	67	81-157 mg/dl	Low
Complement C4 levels	3	10	13-39 mg/dl	Low
Anti Myeloperoxidase	<0.2		<1.0 AI	Negative
Anti Proteinase-3	<0.2		<1.0 AI	Negative
Rheumatoid Factor	<3		<20IU/ml	Negative
Anti GBM	<0.2		<1.0 AI	Negative

**Figure 4:**
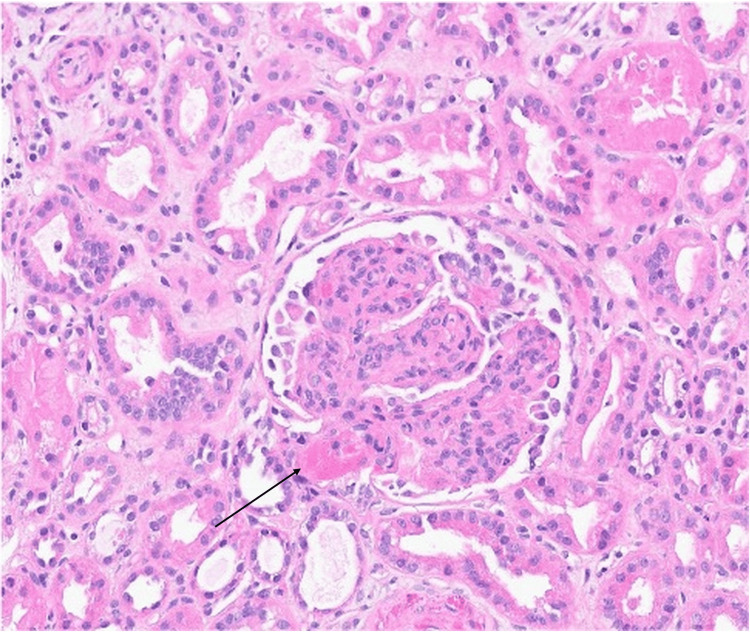
Right Renal Biopsy Demonstrating Fibrin Thrombi. Hematoxylin and eosin (H&E)-stained sections (original magnification x400) from a right renal biopsy. The arrow indicates a fibrin thrombus within a glomerular capillary loop. These findings are consistent with thrombotic microangiopathy (TMA).

Autoimmune hemolytic anemia (AIHA) was initially considered, but a direct antiglobulin test (DAT) was negative. Due to high clinical suspicion, an enhanced DAT was performed and revealed a weakly positive anti-IgG of unclear significance. Concern for renal involvement and an atypical clinical presentation prompted a renal biopsy on hospital day 3, which demonstrated Class IV lupus nephritis with immune complex-mediated membranoproliferative glomerulonephritis (MPGN) and thrombotic microaneurysms - findings concerning for cmTMA (refer to Figure 4 for renal biopsy). 

The patient was initially treated with pulse-dose corticosteroids - 1 g IV methylprednisolone daily for three days. After briefly being transferred to the medicine floors, he required ICU readmission due to acute hypoxic respiratory failure (AHRF) and fluid overload necessitating continuous renal replacement therapy (CRRT), and later transitioned to intermittent hemodialysis (HD). There was additional concern for sepsis and steroid-induced delirium. Following the steroid pulse, a gradual taper was initiated, and IV cyclophosphamide (500 mg) was administered on day six per the EuroLupus protocol, with continued dosing every two weeks. 

**Figure 5:**
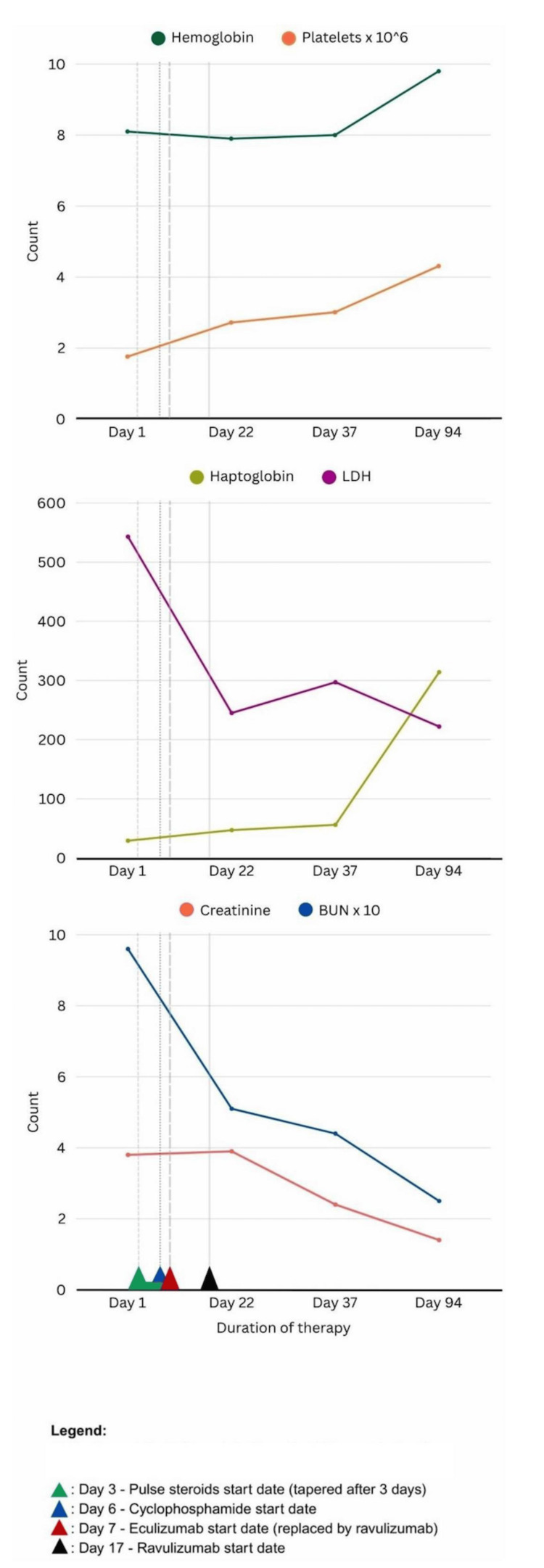
Laboratory trends following initiation of therapy. Units: creatinine (Cr): mg/dL; blood urea nitrogen (BUN): mg/dL; hemoglobin (Hg): g/L; platelet (PLT): count/microliter; haptoglobin: mg/dL; lactate dehydrogenase (LDH): IU/L

Following the meningococcal vaccination series (Menveo) and antibiotic coverage, on day seven, complement blockade was initiated with 900 mg IV eculizumab weekly to treat renal TMAs. This was later transitioned to ravulizumab 2700 mg IV on day 17 for longer-interval dosing (every eight weeks). Following initiation of therapy, hematologic parameters steadily improved: platelet counts remained stable, LDH levels declined, and haptoglobin became detectable by day 22 (refer to Figure 5 for laboratory trends following therapy initiation). Genetic testing for abnormalities in the complement system revealed no known pathogenic variants, although a variant of uncertain significance (VUS) in ADAMTS13 was identified which was not considered clinically actionable.

## Discussion

Complement-mediated TMA can occur in the context of genetic or acquired defects in complement proteins (primary cmTMA) or in association with other disease processes like autoimmunity, medications, malignancy, and infection (secondary cmTMA) ​[3]. Renally restricted TMA has most frequently been found to be secondary to rheumatological disorders or medication use ​[5]. A large retrospective study of 77 patients who were recruited from the Limburg Renal Registry aimed to compare kidney-limited and systemic thrombotic microangiopathies. Based on the genetic complement profile, patients were classified into three groups: those with definite complement dysregulation (definite C-TMA), probable C-TMA, and TMA with normal complement regulation (nC-TMA). Fifty-one (66%) of the 77 patients had renally restricted TMA. The incidence of end-stage kidney disease at 12 months was higher in definite C-TMA (57%) and probable C-TMA (29%) compared to nC-TMA (12%). Patients with definite C-TMA and probable C-TMA also had a higher likelihood of achieving improved kidney response with eculizumab compared to nC-TMA. This underscores the need to screen patients with renal TMAs for complement dysregulation [6]. 

TMA in SLE is associated with endothelial dysfunction, which may be related to complement dysregulation as seen in our patient. Other mechanisms are autoantibody-mediated endothelial injury in antiphospholipid syndrome, microvasculature platelet aggregation in TTP-hemolytic uremic syndrome (HUS), or drug-induced endothelial toxicity. The diagnosis of TMA in SLE is often challenging because of the similarity in clinical presentation with anemia, thrombocytopenia, renal involvement, neurological deficits, and fever. This is especially true when systemic signs like thrombocytopenia are minimal or absent, and lesions are renally limited. Therefore, pathological diagnosis is considered the “gold standard” in establishing the diagnosis. Complement testing may be considered in biopsy-proven TMA; however, its utility should be determined on an individual basis​ [7].

According to the study by Di Song et al., out of a cohort of 148 patients with lupus nephritis, 36 patients were pathologically diagnosed with having renal-TMAs. Twenty-nine of those patients had isolated renal TMAs, while the remainder had associated TTP-HUS, APS, malignant hypertension and scleroderma. It was found that the renal-TMA group had poorer outcomes compared to the non-renal TMA group. Renal TMA was an independent risk factor for poor renal outcomes in patients with lupus nephritis. Of the 36 patients, 52.7% of patients with C4d glomerular deposition and nearly half of the patients with decreased serum complement factor H had poorer outcomes​ [7]. The degree of microthrombi involvement within glomeruli is correlated to the intensity of Cd4 staining, suggesting complement dysregulation to be a key mechanism of injury [8,9].

Low C3 and C4 complement levels are commonly associated with LN, suggesting the inappropriate activation of both the classical and alternative pathways of complement in the development of TMA in these patients [4]. Although, in approximately 60% of cmTMA patients, all complement levels are normal, giving rise to debate regarding the utility of these tests. Despite this, commercial kits are available for the quantification of C5b-9, which is commonly performed in the management of cmTMA. The main utility for complement serology can be found in monitoring C5 blockade in patients receiving complement-directed therapy ​[10].  

The use of terminal complement inhibitors like eculizumab and ravulizumab has been found to be effective in TMA in LN, with hematological parameters improving in one to two weeks and renal function improving over months ​[4].​ A prospective study to measure the efficacy of eculizumab in patients with cmTMA was conducted in two-phase trials. In the first trial, 17 patients with low platelet counts and renal damage demonstrated improvement in platelet counts. In the second trial, 20 patients with renal damage but no decrease of platelet count more than 25% during eight weeks of plasma exchange were recruited. This group demonstrated endpoints with a thrombotic microangiopathic event-free status. Eculizumab was associated with continued, time-dependent improvement in the glomerular filtration rate (GFR) in both groups, with earlier intervention being associated with improved clinical outcomes [11].   

## Conclusions

This case underscores a diagnostically challenging and underrecognized manifestation of thrombotic microangiopathy: complement-mediated TMA with predominant renal involvement in a patient with lupus nephritis. In the absence of classic systemic features such as thrombocytopenia, diagnosis relied on histopathological confirmation, highlighting the importance of maintaining a high index of suspicion in declining patients with lupus nephritis despite standard therapy. Complement level screening may be performed in addition to biopsy, though this is determined on an individual basis. Our patient demonstrated a favorable hematologic and renal response to terminal complement inhibition with eculizumab and ravulizumab, reinforcing the growing evidence for complement blockade in secondary cm-TMAs, even when majorly involving a single organ. As renal cm-TMA may portend worse renal outcomes and be refractory to conventional immunosuppression, early recognition and tailored therapy could be critical. Further studies are needed to define optimal diagnostic strategies, treatment timing, and long-term outcomes in this emerging clinical phenotype.
